# Investigating Learning Effects Through the Implementation of Teledermatology Consultations Among General Practitioners in Germany: Mixed Methods Process Evaluation

**DOI:** 10.2196/65915

**Published:** 2025-09-10

**Authors:** Andreas Polanc, Inka Roesel, Elke Feil, Peter Martus, Stefanie Joos, Roland Koch

**Affiliations:** 1 Institute for General Practice and Interprofessional Care, University Hospital Tübingen, Osianderstraße 5, Tübingen, 72076, Germany, 49 70712985285; 2 Institute for Clinical Epidemiology and Applied Biometry, University Hospital Tübingen, Tübingen, Germany

**Keywords:** telemedicine, teledermatology, teleconsultation, experiential learning, competence gain, general practice, primary care, competence, learning effects, consultations, mixed methods, general practitioners, Germany

## Abstract

**Background:**

The increasing prevalence of dermatological diseases will pose a growing challenge to the health care system and, in particular, to general practitioners (GPs) as the first point of contact for these patients. In many countries, primary care physicians are supported by teledermatology services.

**Objective:**

The aim of this study was to detect learning effects and gains among GPs through teledermatology consultations (TCs) in daily practice.

**Methods:**

As part of a mixed methods study embedded in a cluster-randomized controlled trial (TeleDerm), a full survey and semiguided face-to-face interviews were conducted among GPs of participating intervention practices using the telemedicine approach. A TC assessment tool (TC-AT) was developed to evaluate the quality of clinical data and images of TCs conducted during the run-in and intervention phases, with a score ranging from 0 (lowest quality) to 10 (highest quality). Mixed methods analysis triangulated qualitative content analysis, survey data with a growth curve model calculated from TC-AT data, comparing subjective experiences of GPs with objective process data.

**Results:**

A total of 487 TCs of 33 practices were analyzed. Questionnaires from n=46 GPs (practice-level response rate: 69.9%) were included in the quantitative analysis. Two-thirds of the GPs (n=31; 67.4%) in the written survey rated the TCs as helpful for differential diagnosis and treatment management. Improved self-reported confidence in diagnosing skin diseases due to the timely clinical feedback from dermatologists was reported by more than half of the responding GPs (n=25; 54.3%). In the interviews (n=13), teleconsultations were mainly seen as a learning opportunity by the GPs. Regarding the quality of TCs, a mean TC-AT score of 7.4 (SD 1.7, range 0‐10) was observed. In the growth curve model, a simple linear time trend provided the best fit to the TC-AT score trajectory across the observed study period. A significant time * TC-AT start score interaction was found (*F*_452_=30.66, *P*<.001). While regardless of the initial TC-AT score, repeated TCs lead to process quality improvements over time, post hoc probing of the TC-AT start score as a moderator of the learning effect over time revealed the highest improvements among GP practices with a lower initial TC-AT score (−1 SD: standardized slope=0.59, *P*<.001; mean: standardized slope=0.38, *P*<.001; +1 SD: standardized slope=0.18, *P*<.001).

**Conclusions:**

TCs have been shown to be an effective method of education for GPs in terms of “learning on the job” in daily practice. The telemedicine approach seems to be an easily implementable and effective tool to support continuing medical education in the field of dermatology. Strategies could be developed to train GPs and medical students in the use of TC to adequately prepare them for the increasing technological demands of their future profession in primary care.

## Introduction

### Background

Demographic and environmental factors, climate-related health risks, and lifestyle changes will contribute to a significant increase in skin diseases in the near future, placing a particular burden on health care systems [1-7]. General practitioners (GPs) as the first point of contact for patients with skin conditions play a crucial role in the early detection, accurate diagnosis, and effective management of skin diseases [8]. The care of patients with skin lesions is becoming increasingly difficult due to the growing shortage of specialists, especially in underserved areas.

Teledermatology consultations (TCs) offer an opportunity to alleviate the escalating demand for dermatology services [9-11]. International studies have shown that the telemedicine approach is as effective as traditional medical referrals in reducing costs, enhancing care coordination, and improving patients’ quality of life [12-21].

In addition to the aforementioned benefits of TC for patient care, international studies have reported an increase in confidence and learning effects among GPs through TC with their dermatology colleagues [8,15,18,22-28]. Learning effects were achieved, for example, by refreshing old knowledge, sharing new therapeutic concepts, or exchanging difficult cases that were considered essential for their daily work [15,29]. It has been shown that the percentage of correctly prediagnosed cases by GPs increased through TC [8].

Studies have demonstrated teledermatology (TD) to be an effective educational method and an opportunity for dermatologists’ and medical students’ professional development [30-34]. Lee et al [10] recommended TD as an educational tool for health care providers to interact with for diagnostic support and guidance, especially if they lack specialized dermatology training. In a systematic scoping review of educational programs on melanoma diagnosis for GPs, only 16 of 31 educational interventions included training in dermoscopic diagnosis. Hereof, only one RCT study included TD feedback [35]. To date, little is known about the competence gains, learning effects, and skills acquired by GPs through TC in general practice.

Among these competencies, communication between specialists and primary care is of paramount importance [36]. In a cross-sectional survey by Scaioli et al [37] of more than 7100 GPs in 34 OECD (Organisation for Economic Co-operation and Development) countries on GP-specialist communication in the referral process, the extent and intensity of communication between GPs and medical specialists in a country where a gatekeeping system exists is associated with the organization of the primary health care system. Especially in societies without a mandatory gatekeeping system, as in Germany, there is a higher risk of poor communication between different levels of care [36,37]. In addition, the high workload, the shortage of doctors, and the associated time pressure on doctors may also have a negative impact on communication behavior. According to OECD statistics, Germany had an average of 9.8 doctor-patient contacts per capita in 2019, compared with an OECD average of 6.8 doctor contacts per capita [38].

Despite the need for a more direct exchange between specialists and GPs, written communication still remains the most common form of interdisciplinary exchange between specialists and primary care [36]. Rübsam et al [] [39] identified a need for optimization of interdisciplinary exchange with dermatologists among German GPs, mainly because written feedback from dermatologists was often missing or incomplete, which led to poor communication with dermatology colleagues and also made it very difficult for GPs to pursue or even effectively implement appropriate diagnoses and therapies and deprived them of the opportunity to receive further training based on written feedback 39.

The use of health information technology and a closer collaboration between GPs and specialists have been shown to improve communication [36]. For example, teledermatology has proven to be a useful tool to support direct communication between dermatologists and GPs [23].

While communication via telemedicine was already an integral part of patient care in many European countries, in Germany, liability, professional and data protection regulations, a heterogeneous organization of regional health care due to federalism, and a rather conservative attitude of users have made it difficult to introduce health technology on a large scale [40-43]. It was not until the amendment of the Medical Practitioners’ Code (MBO-Ä) in 2017 and the new regulation of exclusive telemedical treatment in the Professional Code of the Medical Association of Baden-Württemberg in June 2020 that the way was paved for the implementation of telemedicine services beyond pilot projects in Baden-Württemberg, about 20 years later than in other European countries [42,44].

Apart from the regulatory framework, it has been proven that adequate reimbursement for teledermatology services plays a critical role in provider participation and the sustainability of such programs [45]. With the amendment of the reimbursement regulation in the statutory insurance on October 1, 2020, a TC request as well as the evaluation of the TC by a medical specialist from then on can be reimbursed [46]. The latter includes not only the assessment of the medical problem, but also a written report to the GP who requested the TC.

Given the persistent deficiencies in interdisciplinary communication, the integration of asynchronous store-and-forward (SaF) TC approach seems to be a promising way to close this communication gap, while at the same time providing German GPs with an on-the-job learning opportunity.

### Research Aim and Questions

There is little evidence that TCs lead to learning effects in “real-world” GP settings. The aim of our substudy within the randomized controlled TeleDerm trial was thus to identify learning effects in GPs, defined as subjective reporting of competency gains in combination with objective improvement in process quality. The accompanying mixed methods process evaluation addressed the following research questions: (1) to what extent can the SaF approach be considered an effective training tool for GPs in daily practice in the sense of “learning on the job”, (2) what is the impact of case-based interdisciplinary communication through direct feedback using the SaF approach on the competence and confidence of GPs in the diagnosis of patients with skin complaints and in the assessment of dermatologic diseases, and (3) does the frequency of TC requests in combination with direct feedback from dermatologists support an improvement in the process quality improvement in TCs of GPs over time, indicating competency gains? Based on the results, strategies for the use of TC in GP training and medical education, and for the implementation in primary health care can be derived. The results from this study may benefit health care researchers and persons involved in the continuous professional development of GPs, especially in training scenarios in the context of day-to-day clinical practice.

## Methods

### Setting and Study Design

**Figure 1.**
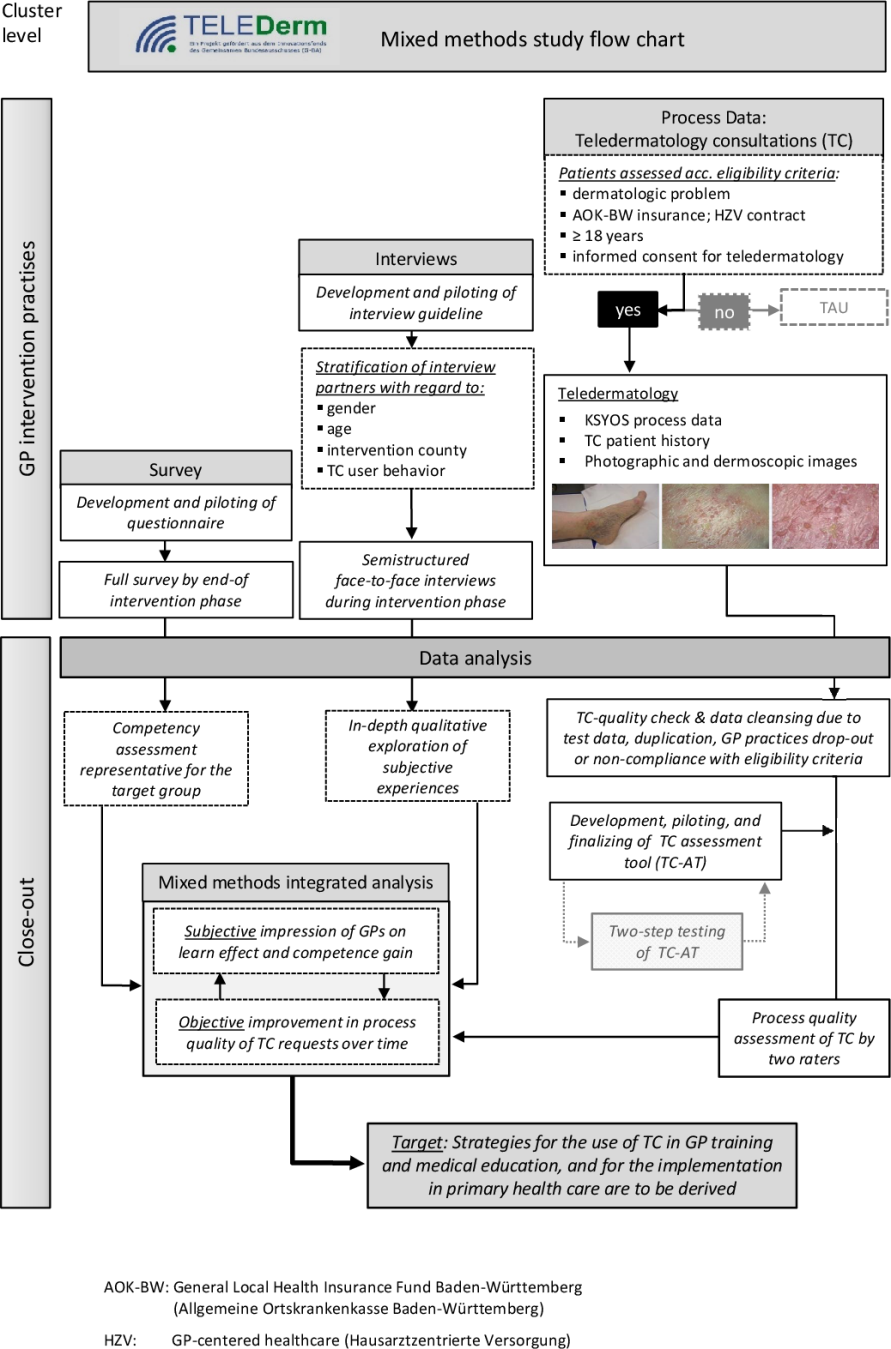
Mixed methods study flow chart. GP: general practitioner; TC: teledermatology consultations; TC-AT: teledermatology consultation assessment tool.

TeleDerm was designed as a 2-arm, cluster-randomized confirmatory trial with an accompanying mixed methods process evaluation. It was conducted from 2017 to 2020 in 4 rural and semirural intervention counties in the German federal state of Baden-Württemberg [42,47]. Inclusion criterion for all GP intervention practices (IPs) and patients with skin complaints aged 18 years and older was their participation within the GP-centered health care program of the General Local Health Insurance Fund Baden-Württemberg (AOK-BW). All patients who gave written consent to participate in the TeleDerm trial underwent TC, while patients who refused TC were treated as usual. Based on standardized case documentation, the TC system within our study provided asynchronous (SaF) recording, transmission, and reporting of the patient history (eg, medical history, differential diagnosis, medications, therapy, information on complaints, duration, and changes in shape, color, or size of the lesion). In addition, images of the affected skin area were taken by the primary care physician using a digital camera. The assessment of the TC was to be completed within 48 hours. Via a secure web interface, the dermatologists provided direct, timely, and comprehensive case-based feedback including *ICD-10* (*International Statistical Classification of Diseases and Related Health Problems, 10th Revision*) code and management advice in free text to the requesting GP. In case of further questions from either the GP or the dermatologist, a one-time “question loop” was set up. This meant that when a TC request was made, either the GP or the dermatologist had a one-off opportunity to communicate directly with their medical colleague via a secure web interface to discuss and clarify any outstanding issues relating to the specific TC request. Multimedia Appendix 1 shows the detailed SaF process steps for TC requests in the TeleDerm study. Further information on the study design can be found in the peer-reviewed TeleDerm study protocol [47]. A detailed overview of the mixed methods flow chart within our study is depicted in Figure 1.

### Survey

The paper-based questionnaire was developed based on literature and informal interdisciplinary exchange during the preparatory phase of the study and covers the categories of learning effects and competence gain with 9 questions. Additional free text entries were permitted in the questionnaire. In February 2019, the questionnaire was piloted (n=5) with representatives of the target groups using the think-aloud method, and between May and June 2019, the survey was conducted in the participating GP practices (n=46 IP). During its development, the questionnaire was presented several times in an interprofessional plenary session at the Quantitative Methods Research Workshop at the University Hospital of Tübingen and discussed methodologically. Descriptive statistics were calculated by AP using SPSS Statistics version 28.0.0.0 (IBM Corp.). Categorical variables were expressed as frequencies and percentages. A chi-square test (*χ*^2^) was performed to compare user behavior (frequent users vs infrequent users) and learning effect. Free text responses were categorized thematically, using the categories and subcategories developed in 2.3 as a template. Quotes from the free text fields of the survey in the article are marked with the abbreviation “QuesTC.” Missing values are excluded from further analysis.

### Interviews

Semistructured face-to-face interviews with GPs explored both the impact on interprofessional collaboration and communication between GPs and dermatologists, as well as the competence gain of GPs through feedback from dermatologists. Before piloting the interview guideline with representatives of the target group, the guideline was presented and methodologically discussed in an interprofessional plenary session at the Qualitative Methods Research Workshop at the University Hospital of Tübingen. The interview guideline was piloted by AP in February 2019 (n=5) using the think-aloud method [48]. The participants were asked to give their interpretation of the questions and to explain their answers. According to their feedback, no changes to the interview guideline were necessary.

A stratified selection of interviewees was made according to age (young, middle-aged, and older), gender (male, female), county of origin, and TC use behavior (nonresponder, occasional user, and frequent user). An initial sample size of n=20 interviews with GPs was determined using Malterud 5-dimensional approach of “information power”. The dimensions comprise study aim (exploratory), sample specificity (high), established theory (experiential learning [49]), quality of dialogue (expected: mediocre, which means low information density in the transcripts), and analysis strategy [50]. Should the estimated sample size prove insufficient during the analysis, we reserve the right to recruit additional participants. Within the mixed methods evaluation, semistructured face-to-face interviews (AP and EF) were conducted starting March 2019, after informed consent to participate was obtained. Digital recordings of the interviews were transcribed verbatim.

Analysis started with an initial reading of the transcripts in April 2019 to get to know the material and assess the quality of dialogue. Based on this initial reading, we determined in May 2015 that no further information would be obtained through additional interviews due to a high degree of repetition in the interview transcripts and a better dialogue quality than expected. At that point, recruitment to additional interviews was stopped at 13 interviews, and we began with the qualitative analysis.

For qualitative analysis, we assumed a constructivist paradigm that individuals make sense of their subjective reality through narration [51]. Health care professionals learn continuously based on concrete experience [49,52-55]. Qualitative analysis was performed with software support using MaxQDA (VERBI Software GmbH; version 20.1.0) according to Mayring and Fenzl [56]. First, an initial coding frame was built deductively based on the questions of the interview guideline. Both authors (AP and EF) independently applied the coding frame to the same 3 transcripts to assess consistency and reach a shared understanding of how the codes were to be applied. According to the principles of qualitative content analysis, the coding frame was organized into main and subcategories and inductively extended by systematically paraphrasing and generalizing text segments, followed by the formulation of new categories based on recurring content and consensus between coders. Under the supervision of RK, the researchers (AP and EF) iteratively compared new data against existing codes or categories. This ongoing process allowed us to refine and validate the coding frame, ensuring it reflects the evolving understanding of the data. The refined coding frame was then used by AP and EF to code the remaining transcripts, adding and refining categories whenever new information had to be integrated. The coding frame and initial results were also presented and discussed in an interprofessional plenary session at the Qualitative Methods Research Workshop at the University Hospital of Tübingen, in an initial stage as well as the final frame with its detailed categories and subcategories. Quotes from the interviews in the article are marked with the abbreviation “Int.”

### Development and Reliability of a TC Assessment Tool

Dermatologists need comprehensive clinical data and high-quality images for effective, efficient TC. Especially image quality is crucial for ensuring the same quality of care as a face-to-face consultation [57-59]. A TC assessment tool (TC-AT) was developed to provide a retrospective evaluation of the quality of the TC process. In addition to assessing the quality of the uploaded images, the tool was designed to assess whether the dermatologist was provided with all of the patient information necessary for diagnosis, a detailed description of the dermatologic changes, and the diagnostic question. The results of the AC-TC assessments should show the extent to which the participating GPs were able to perform the relevant process steps in the same way throughout the study and to “deliver” the information relevant to the dermatologist’s diagnosis with regard to improvement over time.

Referring to the training concept developed for the participating GPs in the TeleDerm study and the recommendations of the TC guidelines of the German Dermatological Society (DDG) and the Federal Association of German Dermatologists (BVDD) [60], 2 of the authors (AP and RK) developed an assessment tool focusing on the process quality of clinical data and images for SaF TC. Based on the evaluation of randomly selected TCs by 2 raters (AP and RK), the TC-AT was developed and evaluated within a 2-step process. To ensure the uniformity of the raters’ evaluation and to guarantee the simplicity of the criteria catalog, it was streamlined after the first evaluation and the criteria were formulated more specifically (Step 1). Both the pairwise Cohen kappa statistic for single item TC assessment scores (к=0.852; 95% CI 0.776‐0.928) as well as the intraclass correlation coefficient (ICC) for overall item TC assessment scores (ICC=0.871; 95% CI 0.697‐0.945) based on a final assessment of randomly selected TCs (n=24) showed a very good test-retest reliability (Step 2).

Whereas the interrater reliability calculation for assessment of randomly selected TCs (n=24) for single scores was carried out by means of the pairwise Cohen kappa statistic, the reliability concerning the overall TC assessment scores was analyzed by using ICC estimates and their 95% CIs based on a mean-rating (k=2), absolute-agreement, 2-way random-effects model, both times using SPSS Statistics (IBM Corp.; version 28.0.0.0).

The adjusted and finalized qualitative evaluation criteria covered the following aspects: patient history, description of skin area, dynamics of changes, therapy, and medical assessment, as well as the quality of images regarding exposure, contrast, and acuity (Table 1). Each item with respect to clinical data was scored with either 0 (quality criterion not fulfilled) or 1 point (fulfilled). To appropriately weight the importance of the image quality in the evaluation process, image quality was scored from a minimum of 0 to a maximum of 5 points. Item scores were summed up, resulting in an overall TC-AT score range from 0 (poorest quality) to 10 points (highest quality) per TC.

**Table 1.** Teledermatology consultation assessment tool (TC-AT).

Item number	Item name	Criteria	Points
A. Clinical data			
A.1	Patient history^a^	Symptoms and duration of complaints	1
A.2.	Skin area^b^	Description of localization, size, and color	1
A.3.	Dynamic^c^	Changes in size, shape, and color of the affected skin area	1
A.4.	Therapy	Information on medical treatment so far	1
A.5.	Medical assessment	Formulation of a presumptive diagnosis or question for the dermatologist	1
B. Images			
B.1.	Quality of images^d^	Exposure, contrast, and acuity	5
Overall TC assessment score		Maximum points	10

a Patient history: at least 1 out of 2 listed criteria is to be specified

b Skin area: at least 2 out of 3 listed criteria are to be specified, otherwise the item is scored 0 points

c Dynamic: at least 1 out of 3 listed criteria is to be specified

d Quality of images: 3 out of 3 criteria: 5 points (all fulfilled); 2 out of 3 criteria: 4 points (exposure, contrast); 1 of 3 criteria: 3 points (exposure); 0 of 3 criteria: 0 points (nothing fulfilled, not usable)

### Statistical Analysis of TC-AT Score Time Trend

To explore the evolution of TC quality over time, a mixed model was fitted to the TC-AT data. As the total number of TCs per GP practice differed and time points of TCs were unevenly spaced, time was introduced as a continuous variable. In the model building process, linear and quadratic time terms were introduced as fixed effects. Furthermore, the interaction between the starting TC-AT score at baseline and time was assessed. In addition, we investigated whether introducing the overall number of TCs per GP practice as a covariate added significantly to the improvement in model fit. A random intercept and slope for GP practices were added to account for cluster effects. Due to convergence problems, a simple diagonal covariance structure for random effects was chosen. For the residuals, an autoregressive error structure AR(1) was assumed. Model selection was achieved by forward selection: terms were only added when the model significantly improved, as tested with a log-likelihood ratio test. In addition, the Akaike information criterion and Bayesian information criterion were evaluated, deeming models with lower fit criteria as more appropriate.

The final mixed model included time (linear) and a time*baseline TC-AT start score interaction as fixed effects and a random intercept for GP practices. Simple slopes analysis as post hoc probing was conducted to determine the nature of the interaction. We furthermore applied the Johnson-Neyman technique to identify the region of significance. Model assumptions were checked visually and found adequate. A *P* value <.05 (2-sided) was considered statistically significant and adjusted for multiple testing with Bonferroni correction where necessary. All data analyses were implemented in R (version 4.1.3; R Core Team) and R Studio (version 2022.02.1; Posit Software, PBC). Linear mixed models were fitted using the *lme4* [61] and *nlme* [62] packages.

To assess whether the frequency of TC also impacted the subjective impression of competency and confidence gain, the GPs’ user behavior was categorized into frequent users (≥11 TC) or low users (≤10 TC) on the practice level. The cutoff value was based on the median number of teleconsultations per practice. A chi-square test was used to compare the GPs’ user behavior and their subjective learning effect. No expected cell frequencies were below 5.

### Ethical Considerations

The TeleDerm study was approved by the Ethics Review Board of the Eberhard Karls University of Tübingen (Ref. No. 395/2017BO1). Participation in the study was voluntary. Participants were informed of the study’s purpose and their right to withdraw and prohibit the use of their data at any time. They gave written consent to participate in the study, and to collect and to use their data for the study’s purposes.

## Results

### Characteristics of Participants

A total of n=49 GPs participated in the full survey. One GP was excluded from further analysis of the survey because this IP did not conduct any TC. In addition, 2 other GPs were excluded from further analysis because they did not respond to the questions on learning effects and competence gain. Thus, questionnaires from n=46 GPs (response rate of 69.6% on practice level) were included in the further quantitative evaluation.

Table 2 summarizes the main characteristics of the study population at the level of GP practices and health care providers in the full survey of GP intervention practices and in the semistructured interviews with GPs.

**Table 2.** Practice and provider-level characteristics of the study population.

Characteristics	Survey	Semistructured interviews
GPs in total, n (% on GP-practice level)	46 (69.6)	13 (28.3)
Male, n (%)	33 (71.7)	8 (61.5)
Age (years), mean (SD, range)	56.87 (7.963, 37‐75)	59.0 (8.617, 40‐75)
Job experience (years), mean (SD, range)	22.09^a^ (9.273, 1‐38)	Not recorded

a n=1 missing

### Educational Value of TCs

**Figure 2.**
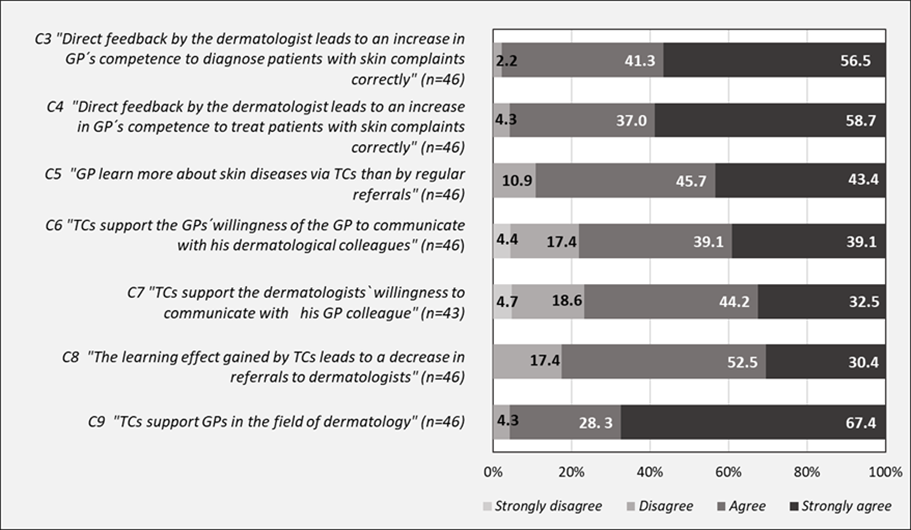
Learning effect (Survey).

According to the survey, 9 out of 10 GPs stated that they learn more about skin conditions through TC than by regular referrals, of which 20 GPs (43.4%) strongly agreed with this item (Figure 2, item C5).

In the interviews, several GPs elaborated that in contrast to normal referrals, TC offers a direct, case-based exchange between specialist and GP:

*In the past, patients were sent to the dermatologist, who examined them and wrote a letter. Today, this feedback from dermatologists is almost non-existent. And that’s a pity because in the meantime we no longer know whether the tentative diagnosis was correct or not, what the dermatologist is doing. So, this learning effect, which we used to have automatically through the report of the specialist, has largely disappeared in the meantime. And this would now be an opportunity to reconnect with this learning effect that we used to have automatically.*

(IntA3-29)

The GPs emphasized the benefit of a timely, direct reporting from the dermatologist for their learning effect, as opposed to regular referrals

*For me, the learning effect is good, simply also because of the quick feedback*

(IntA12-64)

and their therapy recommendations for the further treatment of the GP patients

*We started the therapy on the recommendation of the dermatologists, and it was quite successful*.

(IntA5-24)

In addition, interviewees indicated that personal feedback helps physicians to validate their own findings and thus actively provides an educational impetus for the primary care physicians:

*Sometimes you are confirmed, and sometimes new aspects are added, which you nevertheless include and perhaps treat*.

(IntA7-38)

Despite the many positive effects of direct and personal interaction between GPs and their dermatology colleagues, some respondents were skeptical about the use of telemedicine for communication between the 2 groups. According to the survey results, more than 1 in 5 physicians disagreed with the statement that TCs promote GPs’ willingness to communicate with dermatologists, while 39.1% strongly agreed with this point (item C6). GPs were even more skeptical about the willingness of dermatologists to communicate with GPs via TC. Only one-third (32.5%) of GPs strongly agreed that telemedicine increased dermatologists’ willingness to communicate with GP colleagues, while 23.3% disagreed, including 4.7% who strongly disagreed (Figure 2, item C7).

When asked about the support of GPs by TCs in the field of dermatology, the survey revealed an agreement among the participating physicians about the benefits for their daily practice. Using a 4-point Likert scale, two-thirds of the respondents (67.4%) strongly agreed that TCs support them, while only a small number of respondents (4.3%) strongly disagreed with their colleagues in this regard (Figure 2, item C9).

### Increase in Confidence of Diagnostic and Management Skills in GPs

The GPs were asked about their feeling of learning and competence gain during the TeleDerm project. On a 3-point Likert scale, 54.3% of the GPs reported an improvement in their ability to correctly diagnose patients with skin diseases (item C1). In total, 47.8% of the respondents stated that their competence in performing and assessing dermoscopy in patients with skin diseases had increased during the TeleDerm study (item C2), 77.3% of them were male GPs. One in 2 GPs had not noticed any change in either of these areas.

Interview participants reported that direct interaction between GPs and dermatologists has a positive impact on physicians’ confidence and acquisition of dermatologic knowledge regarding the treatment and management of common skin diseases and lesions.

*You have a tentative diagnosis and now, through the TC, you have the possibility to get confirmation. You gain confidence and that is of course an enormous learning effect*.

(IntA3-29)

In the survey, almost all GPs agreed that the direct feedback from the dermatologists leads to an increase in GPs’ competence to correctly diagnose (Figure 2, item C3) and treat (Figure 2, item C4) patients with skin complaints.

Physicians also see qualitative potential in the learning effect in terms of increased treatment safety, strengthening of their role, and simplification of processes in the care system:

*[The learning effect] of course also offers a safety factor that can be gained for oneself as a therapist and makes many steps simply unnecessary*

(IntA10-34)

This statement was further substantiated by a GP, especially regarding a possible relief of the dermatologists as well as an extension of the approach to other areas of work in family medicine:

*TeleDerm-approach is a good introduction to further telemedicine applications in the classic family doctor/specialist division of labor. There is thus a possibility of avoiding the clogging of the specialist’s schedule with unnecessary consultations*

(QuesTC43-04)

While GPs were overall very positive about the previous items presented, the respondents seemed to be more cautious about the statement that the learning effect gained by TC will lead to a decrease in referrals of patients with skin complaints to dermatologists. Only 30.4% of GPs strongly agreed with this statement, while almost 1 in 5 GPs (17.4%) disagreed with their colleagues (Figure 2, item C8).

### Learning Curve Through Iterative Processes

The number of TC requests during our study had a positive effect on GPs’ subjective competence gain in frequent users. Among GPs who frequently used the service, 64.3% (n=18) reported an increase. The findings of this study, as evidenced by the results of the survey, demonstrated a significance between user behavior and competence in performing and assessing dermoscopy in patients with skin disease, as indicated by the following statistical analysis: *χ*² (1)=7.769, *P*=.005, *φ*=0.411.

Interview participants also reflected on the association between the frequency of TC and GPs’ professional expertise in the field of diagnostic and therapy of patients with skin diseases:

*I would have liked it better if we could have presented many more patients, because the knowledge gain for each of us would have been correspondingly higher*.

(IntA4-27)

Next to repetitive TCs, the use of a dermascope was associated with learning from the GP’s perspective:

*By [.] working with the dermascope, you look at it even more closely [.] and when you get the feedback, you’ve already learned something from it - in case of a repetition, it’s easier to recognize what it was*

(IntA1-47)

### TC Data Flow

Of the 568 TC datasets, 71 TCs had to be excluded due to GP dropouts, duplicates, TC sham datasets, or non-compliance with inclusion criteria.

In addition, 5 nonresponding practices did not conduct any TCs. So far, there is no evidence of when a learning effect can be expected from repeated TC requests. However, since curve fitting requires at least 3 points to derive a trend, only IPs with more than 2 TCs were included in the further analysis of the learning effect. Therefore, n=8 practices with less than 3 TCs per practice during the run-in and intervention phases were excluded, for a total of n=10 TCs. Finally, a total of 33 practices with 487 TCs were included in the subsequent statistical analysis to examine the learning effect over time.

### Learning Effects

The median number of TCs on the practice level was 10 (IQR 6, range 3‐75), and the mean assessment TC-AT score evaluated by both raters reached a value of 7.44 (SD 1.68; median 8, IQR 2, range 0‐10). It has to be pointed out that 3 “*power-user*” IPs were responsible for more than one-third (n=174; 35.72%) of all TC requests during the run-in and the intervention phase. The last TC was conducted 426 days after baseline (ie, after the first TC of the respective IP).

**Figure 3.**
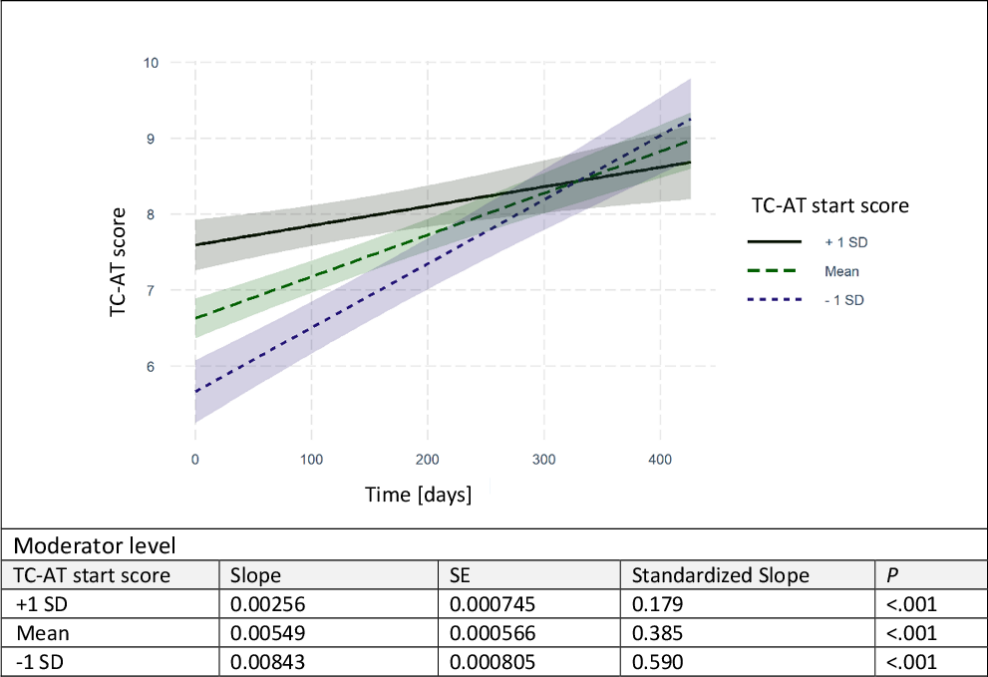
Post hoc probing of the moderation effect of the teledermatology consultation assessment tool start score on the learning effect over time. TC-AT: teledermatology consultation assessment tool.

Results of the final linear mixed model are given in Table 3. A statistically significant time * TC-AT start score interaction was found (*F*_452_=30.663, *P*<.001), indicating that progression of TC quality over time was influenced by the initial TC-AT score. In Figure 3, post hoc probing of the interaction is displayed by simple slopes at the mean (=average TC start score; dashed line) and 1 SD below (=low start score; dotted line) and above the mean TC-AT score level (=high start score; solid line). At all 3 moderator levels, there was a statistically significant positive effect of time on the improvement in TC quality (*P*<.001). The learning process over time was strongest for GPs with a lower-than-average TC-AT start score (standardized slope=0.590) and less pronounced for GP practices starting with a higher-than-average TC-AT start score (standardized slope=0.179). The Johnson-Neyman plot (Multimedia Appendix 2) revealed that the interaction was statistically significant below 249 days.

**Table 3.** Linear mixed model results for the evolvement of the teledermatology consultation assessment tool score over time.

	TC-AT score			
Fixed effects	Β^a^	β^b^	95% CI	*P *value
(Intercept)	3.649	.03	−0.09 to 0.15	<.001
Time	0.014	.38	0.31‐0.46	<.001
TC-AT start score	0.460	.30	0.17‐0.43	<.001
time * TC-AT start score	−0.001	−.21	−0.28 to −0.13	<.001
Random effects				
σ^2^^c^	1.90	—^d^	—	—
τ00^e^	0.16	—	—	—
ICC^f^	0.08	—	—	—
N _practices_	33	—	—	—
Observations	487	—	—	—
Marginal R²	0.252	—	—	—
Conditional R^2^	0.309	—	—	—
AIC^g^	1730.031	—	—	—

a B: unstandardized beta coefficient.

b β: standardized beta.

c σ2: Within-group (residual) variance.

d Not applicable.

e τ00: Between-group variance.

f ICC: intraclass correlation coefficient.

g AIC: Akaike information criterion.

## Discussion

### Principal Results

Our findings show subjective improvements in GPs’ competency paired with observable improvement in TC process quality over time assessed through the TC-AT score. The mixed methods analysis revealed that 2 main aspects contribute to these learning effects: interdisciplinary communication and learning on the job. Both aspects will be discussed in the context of the current research situation in the following section.

### Interdisciplinary Communication Increases GP Expertise

According to our study, timely feedback and direct exchange with specialists on diagnosis, therapy, and treatment options seem to promote learning by validating the GP’s own findings. Thus, it may increase the confidence of physicians in treating patients with skin diseases in daily practice.

As with TeleDerm, German GPs in the study by Rübsam et al [39] criticized the communication with the dermatologists and the lack of written feedback, which meant that the learning effects that GPs had previously gained from the specialists’ reports were lost, making it difficult for GPs to understand and effectively implement an appropriate diagnosis or treatment. Apart from a few critical voices, the majority of our GPs were of the opinion that the TC approach was a supporting tool and a very good opportunity to increase the willingness to communicate and thus to improve the interdisciplinary exchange on both sides in the future. Also, based on the findings of van den Akker et al [23], TC is viewed as a useful tool for interpersonal communication between GPs and dermatologists.

The standardization of TC web templates to ensure the collection of necessary information not only promotes their structured implementation but also provides guidelines for structured interdisciplinary communication between GPs and dermatologists, thus increasing diagnostic and management concordance [25]. This process is particularly supported by the provision of good diagnostic images. According to the results of our main article on the TeleDerm study, 79.8% of the specialists stated that the image quality provided by the GPs during the intervention phase was sufficient for the diagnosis, underlining the importance of this approach [42].

By supporting this interdisciplinary exchange with specialists, the telemedicine approach proves to be a helpful training tool for physicians that can be used in daily practice and at the same time offers the opportunity to close the lamented communication gap between German GPs and dermatologists. Especially the recent changes in the framework conditions in Germany (changes in data protection and the medical code of conduct and the possibility of billing for teleconsultations) may contribute to a sustainable change in communication behavior and stimulate direct exchange not only between GPs and dermatologists but also with other disciplines. Although practitioners frequently perceive standardized forms as a source of double work, this study indicates that structured data processing through such tools may enhance the quality of care by organizing relevant information more effectively.

### On-the-Job Learning in Daily Practice

Practices with initially low TC-AT scores showed the steepest improvement in the quality of TC requests over time, indicating a stronger learning effect in inexperienced practices. The improvement in the process quality of TC requests over time can be explained as a result of this on-the-job learning among GPs [42]. Mohan et al [24] also demonstrated a statistically significant improvement in dermatological knowledge among frequent users of SaF TC among GPs. An effect between the time factor and the frequency of use and repetition in everyday practice is to be expected, especially if the TC requests are also used repeatedly by both sides for refresher and training purposes, for example by discussing differential diagnoses, exchanging alternative treatment approaches, or also in the case of reasons for differences of opinion [15,26]. Frequent use appears to be the most effective way for GPs to build competence with the tools. Educational formats should therefore aim to support the initial adoption phase and help overcome early barriers to use.

Based on the Kolb experiential learning cycle, our results show that frequent use of TC and access to an expert opinion on skin complaints help GPs to reflect on their daily practice. International research supports that TC is a time-saving and effective training tool for GPs and supports continuous learning [15,18,33,34]. The interdisciplinary exchange and communication between GPs and dermatologists could promote the continuous and experiential learning process on many medically relevant aspects—therapy, diagnostics, lesion description, and image quality. This low-level implementation facilitates sustainable and continuous medical education for generalists. The integration of TC into the daily clinical practice of GPs appears to have a positive impact on both perceived and self-reported gains in dermatological knowledge, as well as confidence in diagnostic skills and care management. Furthermore, the feedback and self-assessment by GPs participating in the study indicated that the adoption of TC seems to support improvements in GPs’ professional competence, particularly in the assessment of patients with dermatologic complaints. Our results thus follow the findings of international studies that direct and case-based exchange with specialists provides an educational impetus for GPs by refreshing previously acquired knowledge, but also by sharing new therapeutic concepts, especially when used to discuss differential diagnoses, alternative treatment strategies, or even reasons for disagreement [15,18,25,26]. GP’s reflective practice is supported by iterative, direct feedback by specialists.

In Germany, telemedicine was opened about 2 decades later than in many other European countries. In order to cope with the increasing digitalization of health care, today’s medical students must be prepared for the complex challenges of their future professional life [63]. For this reason, the necessary practice-oriented approaches to digitalization and the teaching of interdisciplinary and intersectoral skills should be included in the compulsory curriculum of medical faculties as a useful combination and should be taught there at an early stage [63,64]. By introducing TC early in medical education, the next generation of practitioners will have already navigated the initial challenges, allowing them to focus more effectively on “learning on the job.”

### Strengths and Limitations

The TeleDerm study is one of the first studies to investigate the effects of “learning on the job” among primary care physicians in the context of dermatology teleconsultations. It should be emphasized that the mixed methods process evaluation is based on 3 different qualitative and quantitative data sources. While the process data collected at the practice level from teleconsultation requests provided a large amount of objectively evaluable data from the “real world” to answer the research question, the full survey allowed a representative assessment of the GPs. The interviews also supported an in-depth qualitative investigation of individual experiences and self-assessment of learning effects and perceived competence gains in relation to dermatological expertise of the target group under investigation. The triangulation of the different data sources thus allowed for a comprehensive analysis and a multifaceted consideration of the research questions from the perspective of the GPs at both group and individual levels.

The operationalization of learning effects as subjective competency gains in combination with objective improvements in process quality is limited. A randomized controlled trial design with the main focus on these learning effects with a stronger emphasis on clinical aspects (eg, by issuing a test of dermatologic skills) would have been preferable but was effectively impossible to implement into the main study.

The TC-AT was developed and tested to evaluate the TCs conducted. All TCs included in the evaluation were assessed and objectively evaluated on the basis of these quality criteria. As a limiting factor in the process evaluation, it should be noted that the evaluation matrix on which the qualitative assessment was based was developed post hoc by the authors. Another limiting factor is that only the formal process indicators collected during the study period were evaluated. A professional evaluation of the medical plausibility, such as a professional evaluation of the diagnosis, therapy, or even the treatment success of the teleconsultations, was not provided for in the study design and was therefore not carried out. Nevertheless, the matrix tool allowed a continuous recording of process and data quality over time, and we were able to describe central aspects of GP’s education through TC based on real-world data.

### Conclusions

The telemedicine approach proves to be an effective training tool for enhancing the diagnostic and therapeutic skills of GPs. The positive learning effects are enabled by direct and timely interprofessional exchange. With the increasing digitalization of our health care system, telemedicine has the potential to enrich medical education as well as training of GPs or specialists. Furthermore, incorporating telemedicine into medical education will ensure that future health care professionals are equipped to meet the evolving needs of modern health care delivery.

## Supplementary material

10.2196/65915 Multimedia Appendix 1 Main process steps for teledermatology requests in the TeleDerm study.

10.2196/65915 Multimedia Appendix 2 Johnson-Neyman plot indicating the size and significance of the slope of the teledermatology consultation assessment tool score across time. Shaded areas indicate 95% CIs.

## Abbreviations

AOK-BW: General Local Health Insurance Fund Baden-Württemberg (Allgemeine Ortskrankenkasse Baden-Württemberg)
BVDD: Federal Association of German Dermatologists
DDG: German Dermatological Society
GP: general practitioner
ICC: intraclass correlation coefficient
*ICD-10*: *International Statistical Classification of Diseases and Related Health Problems, 10th Revision*
IP: intervention practice
OECD: Organisation for Economic Co-operation and Development
SaF: store-and-forward
TC: teledermatology consultation
TC-AT: teledermatology consultation assessment tool
TD: teledermatology
