# Supplementary material for: Investigating Learning Effects Through the Implementation of Teledermatology Consultations Among General Practitioners in Germany: Mixed Methods Process Evaluation
Source: JMIR Med Educ. 2025 Sep 10;11:e65915. doi: 10.2196/65915 (PMC12422744; doi:10.2196/65915)
Supplement: Multimedia Appendix 1 [file mededu-v11-e65915-s001.docx]

During the five-month run-in phase of the TeleDerm study, a SaF-TC system was implemented in 49 IPs, followed by in-house training (hands-on demonstration and supplementary training material). The TC service was provided by KSYOS Telemedish Center (Amsterdam, The Netherlands), a TC service operating in the primary care sector for more than two decades and project partner in our study [14]. Based on standardized case documentation, the SaF-TC system provided asynchronous recording, transmission, and reporting of patient history (e.g., medical history, differential diagnosis, medications, therapy, information on complaints, duration, changes in shape, color, or size of the lesion). In addition, images of the affected skin area were taken either as overview images using a digital camera (SONY DSC-W180; sensor specs: 1/2.3" 10.1MP / 3648 x 2736; lens: 35 - 105mm, f/3.1 - 5.6; 3x optical zoom) or, if necessary, as a high-resolution image using a dermoscope (Dino-Lite DermScope Polarizer MEDL4DW; medical device class 1; 93/42 EEC modified 2007/47/EC; 1280x1024 pixels). To ensure interoperability with the practice's Patient Data Management System (PDMS) via a secure web interface, images and medical history data were uploaded by the physician to the KSYOS server located in Amsterdam, The Netherlands. The server was accessed via https/TLS 2.0 and was therefore encrypted using standard methods. In order to comply with data protection regulations, the transmission of the comprehensive patient history data for the TC request was pseudonymized via a classic encrypted http connection to the KSYOS server.

Each dataset represents a specific treatment case (and thus a specific patient) and is assigned a specific pseudonym when the GP creates the TC case. Theoretically, it would be possible for a patient to present with a different dermatological problem during the intervention phase, resulting in a new TC request (new record).

To ensure a continuous quality of the medical TC evaluation, one of the seven dermatologists was selected by an integrated randomization procedure and informed about the TC request by a notification e-mail. The online evaluation of the TC was to take place within 48 hours. The dermatologists provided the ICD10 code and management advice via free text. A one-time "*question loop*" was set up for further questions from either GP or the dermatologist. This meant that when a TC request was made, either the GP or the dermatologist had a one-off opportunity to communicate directly with their medical colleague via a secure web interface to discuss and clarify any outstanding issues relating to the specific TC request. The system was used by the practices during the 17-month run-in and intervention period, during which teleconsultations were conducted when indicated.
