# Supplementary figures and images for: Investigating Learning Effects Through the Implementation of Teledermatology Consultations Among General Practitioners in Germany: Mixed Methods Process Evaluation
Source: JMIR Med Educ. 2025 Sep 10;11:e65915. doi: 10.2196/65915 (PMC12422744; doi:10.2196/65915)

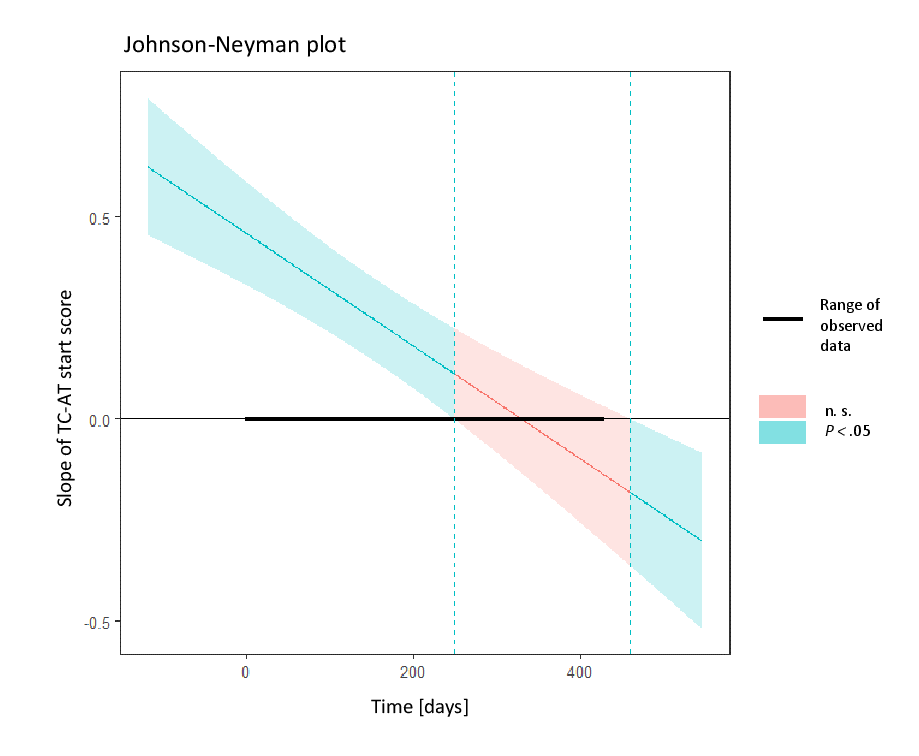

Supplement: Multimedia Appendix 2 [file mededu-v11-e65915-s002.png]
